# Pericardial Effusion as an Early Manifestation of Myeloperoxidase (MPO)-Positive Antineutrophil Cytoplasmic Antibody (ANCA)-Associated Vasculitis With Eosinophilia

**DOI:** 10.7759/cureus.95426

**Published:** 2025-10-26

**Authors:** Amol Karkhanis, Moemen Hasaballah, Mohamed Elsayed, Hassan Rizwan, Anees S Butt, Mohamed Abdulmajeed

**Affiliations:** 1 General Internal Medicine, Luton and Dunstable University Hospital, Luton, GBR; 2 General Internal Medicine, Addenbrooke's Hospital, Cambridge University Hospitals NHS Foundation Trust, Cambridge, GBR; 3 Medicine, Divisional Headquarters - Mirpur Azad Jammu and Kashmir (AJK), New Mirpur City, PAK

**Keywords:** anca-associated vasculitis, churg strauss, pericardial effusion, small vessel vasculitis, steroids

## Abstract

Antineutrophil cytoplasmic antibody (ANCA)-associated vasculitis (AAV) is a rare but life-threatening autoimmune disease that primarily targets the kidneys and lungs. Cardiac involvement is far less common, and when it does occur, it is often overlooked until late in the disease course. We describe an elderly man who presented with acute breathlessness, pericardial effusion, eosinophilia, and worsening renal function. Initial management of pneumonia proved ineffective, and further investigation revealed strongly positive myeloperoxidase (MPO)-ANCA antibodies. The diagnosis of MPO-positive AAV with eosinophilia was established. Following treatment with corticosteroids and rituximab, the patient showed complete resolution of the effusion and significant recovery of renal function. This case highlights the diagnostic challenge of pericardial effusion as an early clue to systemic vasculitis and underscores the importance of early recognition and prompt immunosuppressive therapy.

## Introduction

Antineutrophil cytoplasmic antibody (ANCA)-associated vasculitis (AAV) represents a group of uncommon but severe systemic vasculitides, characterized by inflammation and necrosis of small- to medium-sized blood vessels [1]. The disease spectrum encompasses microscopic polyangiitis (MPA), granulomatosis with polyangiitis (GPA), and eosinophilic GPA (EGPA), each with distinct but sometimes overlapping clinical features [2].

For orientation, GPA is typically characterized by necrotizing granulomatous inflammation affecting the upper and lower respiratory tracts; MPA often presents with rapidly progressive glomerulonephritis and pulmonary capillaritis without granulomatous inflammation; and EGPA classically features asthma, eosinophilia, and systemic vasculitis.

While kidneys and lungs are the principal organs involved in AAV, the heart can also be affected, though this is less frequently reported [3]. Cardiac involvement has been reported in approximately 6-20% of patients with AAV, most commonly manifesting as myocarditis, pericarditis, or conduction abnormalities [4]. Cardiac manifestations range from conduction disturbances and myocarditis to pericarditis and pericardial effusion [4]. Such presentations are clinically important because they carry significant prognostic implications and can complicate an already challenging diagnostic landscape [5]. The presence of eosinophilia, as in our case, adds another layer of complexity. Eosinophilia may point toward EGPA but can also occur in myeloperoxidase (MPO)-positive vasculitis without classical features of EGPA [6]. This overlap can create diagnostic ambiguity, particularly when patients lack hallmark respiratory features such as asthma or chronic rhinosinusitis. Here, we present a case in which a pericardial effusion was the earliest clue to an underlying systemic vasculitis. The narrative illustrates how diagnostic reasoning evolved and how timely immunosuppressive therapy averted irreversible organ damage.

## Case presentation

A 73-year-old man presented with one week of worsening shortness of breath and a productive cough. His medical history was notable for type 2 diabetes mellitus, atrial fibrillation, bronchiectasis, obstructive sleep apnea, prior myopericarditis, and left nephrectomy. The prior episode of myopericarditis occurred three years earlier and was treated conservatively with complete clinical and echocardiographic resolution. There was no evidence of residual pericardial effusion on follow-up imaging, making a chronic effusion unlikely in the current presentation. On admission, he was tachypneic and required 8 L/min of oxygen to maintain oxygen saturation at 93%. His blood pressure was 106/68 mmHg, heart rate 86 bpm, respiratory rate 24/min, and temperature 37.1°C. Physical examination revealed coarse crackles over both lung bases and mild pedal edema, but no jugular venous distension.

**Figure 1:**
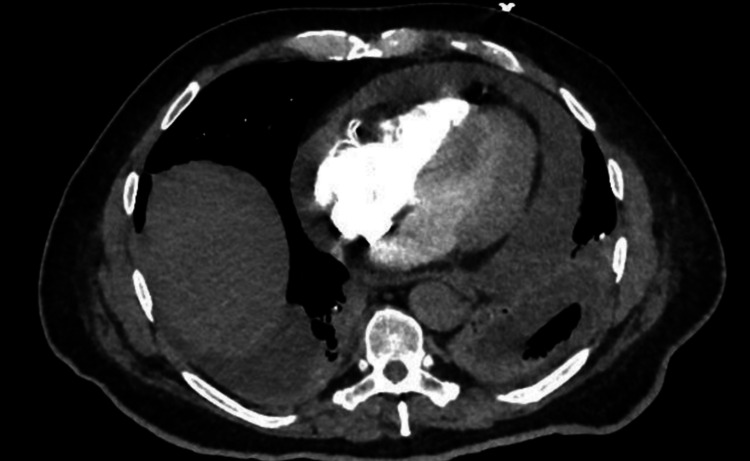
CT chest demonstrating large pericardial effusion CT: computed tomography

Initial laboratory investigations showed raised inflammatory markers, neutrophilia, mild eosinophilia, and acute kidney injury (creatinine 140 µmol/L with a baseline around 95 µmol/L) (Table 1). CT pulmonary angiography excluded pulmonary embolism but demonstrated bilateral consolidation and a large pericardial effusion (Figure 1). Transthoracic echocardiography confirmed a moderate-to-large effusion without signs of tamponade. Cardiac biomarkers, including troponin T (14 ng/L; reference <15 ng/L) and CK-MB (16 U/L; reference <25 U/L), were within normal limits, and there was no serial rise. ECG demonstrated nonspecific T-wave flattening without classical pericarditic ST-segment elevation or PR depression. A chest X-ray showed an enlarged cardiac silhouette consistent with a large pericardial effusion but no pulmonary congestion. These findings supported isolated pericardial involvement rather than myocarditis.

**Table 1:** Laboratory results at presentation WBC: white blood cell, CRP: C-reactive protein

Parameter	Result	Reference range
Hemoglobin	131 g/L	130-170 g/L
WBC count	15.2 ×10⁹/L	4-11 ×10⁹/L
Neutrophils	12.5 ×10⁹/L	2-7.5 ×10⁹/L
Eosinophils	0.5 ×10⁹/L	0.0-0.4 ×10⁹/L
CRP	81 mg/L	<5 mg/L
Creatinine	140 µmol/L (baseline ~95)	60-110 µmol/L
Urea	7.7 mmol/L	2.5-7.8 mmol/L

The patient was initially managed with broad-spectrum antibiotics for presumed pneumonia, but his clinical and biochemical parameters failed to improve. Blood, urine, and sputum cultures remained sterile, and viral panels were negative. Given the ongoing fever, rising creatinine, and progressive eosinophilia (Table 2), an autoimmune cause was suspected.

**Table 2:** Laboratory results one week later WBC: whote blood cell, CRP: C-reactive protein

Parameter	Result	Reference range
Hemoglobin	133 g/L	130-170 g/L
WBC count	15.0 ×10⁹/L	4-11 ×10⁹/L
Neutrophils	8.6 ×10⁹/L	2-7.5 ×10⁹/L
Eosinophils	5.0 ×10⁹/L	0.0-0.4 ×10⁹/L
CRP	99 mg/L	<5 mg/L
Creatinine	383 µmol/L	60-110 µmol/L
Urea	18.6 mmol/L	2.5-7.8 mmol/L

Serology revealed strongly positive MPO-ANCA (>130 U/mL) with negative PR3-ANCA. Bronchoscopy with mucosal biopsy showed inflammatory changes with tissue eosinophilia but no evidence of malignancy. Collectively, these findings established a diagnosis of MPO-positive AAV with eosinophilia.

He was treated with high-dose intravenous methylprednisolone (500 mg daily for three days) followed by an oral prednisolone taper over 12 weeks, in addition to rituximab administered as two 1 g infusions given two weeks apart, following the RAVE protocol.

Following the induction phase, the patient was transitioned to oral prednisolone, which was gradually tapered from 60 mg daily to 5 mg daily over 12 weeks. Rituximab was administered as two 1 g infusions given two weeks apart, with good tolerance and no infusion reactions. He also received adjunctive Pneumocystis jirovecii prophylaxis with co-trimoxazole (480 mg once daily) and gastric protection with a proton pump inhibitor. Blood pressure and glucose were closely monitored throughout steroid therapy. Renal function improved progressively (creatinine decreased from 383 µmol/L to 128 µmol/L), and inflammatory markers normalized by the sixth week. The patient remained stable during follow-up, with no recurrence of effusion on repeat echocardiography at three months.

**Figure 2:**
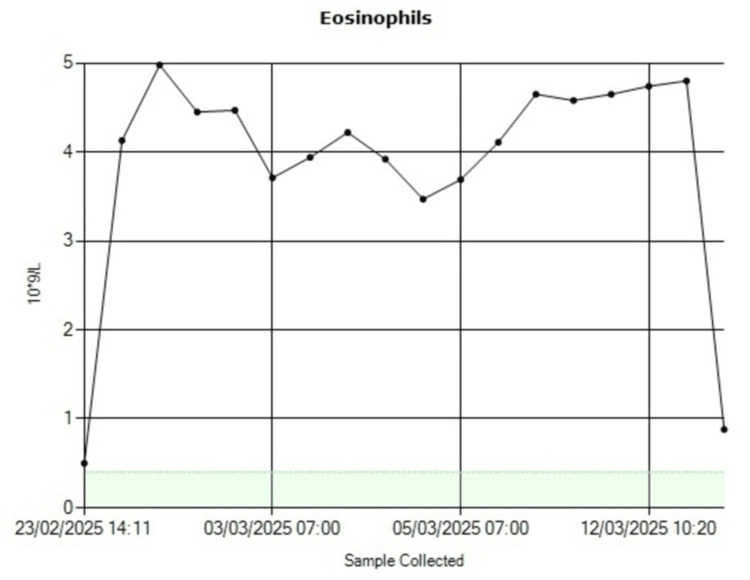
Eosinophils level down trend

The patient’s renal function improved, and inflammatory markers normalized, with eosinophil levels declining progressively to normal values (Figure 2). Echocardiography demonstrated complete resolution of the pericardial effusion. He was discharged home in good condition with outpatient follow-up.

## Discussion

This case highlights the diagnostic challenge posed by atypical presentations of AAV. While renal and pulmonary features dominate most case series [2], cardiac manifestations, particularly pericardial effusion, are rare but important [3,7]. The prevalence of cardiac involvement in AAV ranges from 6% to 20%, with myocarditis and pericarditis being the most common manifestations [3,7]. Pericardial effusion is less common and, when it occurs, is often mistaken for infection, malignancy, or heart failure [4]. Previous reports have documented cases progressing to tamponade, underscoring the seriousness of this complication [5]. In our patient, the effusion was large but hemodynamically stable, providing a vital clue before catastrophic deterioration occurred. The marked eosinophilia initially led the differential toward a diagnosis of EGPA. However, EGPA is typically characterized by asthma, sinus disease, and pulmonary infiltrates [6]. The absence of these features, coupled with high MPO-ANCA titers, favored MPO-positive AAV with eosinophilia rather than classical EGPA [8]. This overlap demonstrates the limitations of rigid disease classification and reminds clinicians to interpret laboratory findings within the broader clinical context. This case illustrates how physicians must balance probabilities. The patient’s presentation with fever, consolidation, and effusion strongly suggested infection, and empiric antibiotics were appropriate. Yet persistence of inflammation, renal dysfunction, and rising eosinophils warranted reconsideration. The eventual pivot to autoimmune testing demonstrates the iterative nature of diagnostic reasoning in real-world practice.

Standard induction therapy for AAV involves corticosteroids combined with either cyclophosphamide or rituximab [8]. The pivotal RAVE trial demonstrated that rituximab is non-inferior to cyclophosphamide and may be more effective in relapsing disease [9]. Our patient’s favorable response to steroids followed by rituximab aligns with this evidence. Beyond acute management, relapse prevention remains a significant challenge, with long-term maintenance using rituximab or azathioprine shown to reduce recurrence [10,11]. Long-term survival in AAV has improved substantially with modern immunosuppression, but mortality remains higher than in the general population [10]. Renal involvement is the strongest predictor of poor outcome, while cardiac manifestations add further risk [12]. Early recognition, therefore, is crucial, not only to reverse acute pathology but also to alter long-term prognosis.

This case reinforces the importance of maintaining diagnostic flexibility. When faced with a patient whose course does not follow the expected trajectory, clinicians must be prepared to step back, reconsider, and broaden their differential diagnosis. In retrospect, the pericardial effusion represented the earliest manifestation of the underlying vasculitic process, preceding the development of overt renal and hematologic features. Recognizing that eosinophilia was not solely attributable to infection but could also signal vasculitis was pivotal.

## Conclusions

Pericardial effusion can rarely represent the initial presentation of MPO-positive AAV with eosinophilia. Its occurrence in the context of systemic inflammation, eosinophilia, and acute kidney injury should prompt consideration of vasculitis, particularly when infection is excluded. Timely initiation of immunosuppressive therapy is essential to prevent irreversible organ damage. This case illustrates how careful observation, diagnostic adaptability, and early multidisciplinary intervention can dramatically alter the patient’s outcome.
